# Pregnancy maternal fetal outcomes among pregnancies complicated with atrioventricular block

**DOI:** 10.1186/s12884-022-04650-x

**Published:** 2022-04-10

**Authors:** Kana Wang, Junguo Xin, Guiqiong Huang, Xiaodong Wang, Haiyan Yu

**Affiliations:** 1 Department of Obstetrics and Gynecology, West China Second University Hospital, Sichuan University, No. 20, 3rd section, South Renmin Road, Chengdu, 610041 Sichuan China; 2 Key Laboratory of Birth Defects and Related Diseases of Women and Children, Ministry of Education, Sichuan University, No. 20, 3rd section, South Renmin Road, Chengdu, 610041 Sichuan China; 3 School of Public Heath, Chengdu Medical College, Chengdu, China

**Keywords:** Atrioventricular block, Cardiac arrhythmias, Gestation, Pacemaker

## Abstract

**Background:**

Atrioventricular block (AVB) during pregnancy is rare. Case study for pregnancy with AVB have been reported but a consensus guideline for peripartum management has not been established. This study aimed to investigate cardiac and obstetric complications and outcomes in our pregnant women with AVB and share our management experience.

**Methods:**

This was a retrospective study. We reviewed a total of 74 pregnant women with AVB who delivered at our tertiary care center in the past 10 years. The patients were categorized into four groups according to the degree of block. The data were analyzed and compared among the four groups of patients.

**Results:**

Regarding the cardiac complications, the cardiac function level showed significant difference among patient groups. The higher NYHA class were observed in patients with higher degree AVB. Pacemaker was placed before delivery in 32/33 patients with III° AVB, 8/25 patients with II° AVB, and 0/16 patient with I° AVB. Other types of arrhythmias except AVB were present in all groups of patients but more frequently observed in type I patients with II° AVB. No other heart abnormalities were observed among the patient groups. Obstetric complications were found in 21 women (28.4%), including premature labor, premature rupture of membranes (PROM), gestational diabetes mellitus (GDM), preeclampsia, etc. The incidence rate of fetal cardiac abnormalities was 6.58%. But no statistical difference was detected among four groups of patients for fetal and maternal complications and fetal cardiac abnormalities (*P*>0.05). Caesarean section was performed more in patients with high-degree AVB than in patients with low-degree AVB. No maternal or neonatal death in our cases.

**Conclusions:**

Most women with AVB could achieve successful pregnancy and delivery. Patients with II° AVB type II and III° AVB should be monitored vigilantly during pregnancy and post-partum. Temporary pacing before delivery appeared to be beneficial for women with III°AVB, and accurate diagnosis and care by a multidisciplinary team was recommended.

## Background

As a serious cardiac arrhythmia, atrioventricular block (AVB) is rarely observed in women during pregnancy. This type of arrhythmia is featured by the loss of the regular function of the cardiac electroconductive pathways through the atrioventricular node (AV node). Without normal conduction through the AV node to ventricle, the sinoatrial node (SA node) cannot control the heart rate, and cardiac output can diminish secondary to the loss of coordinated contraction of the atria and the ventricles [1, 2]. According to the grade of block, AVB was defined at first-degree (I° AVB), second-degree (II° AVB, further divided into two types: Mobitz type I and type II), and third-degree (III° AVB). The third-degree AV block indicates a complete loss of communication between the atria and the ventricles. Some patients may not show symptom and are accidently diagnosed during electrocardiography (ECG) examination for other purposes. Some patients may have a variety of symptoms such as dizziness, weakness, syncope, and even congestive heart failure. These symptoms may worsen with the increase in hemodynamic burden during pregnancy [3, 4]. While I° AVB may be well tolerated during pregnancy, high-grade AVB may increase the risk for both cardiovascular and obstetric complications, which may pose challenge to the treating team. Currently, there is not an established consensus for the peripartum management in patients with high-grade AVB [5, 6]. There are only case reports or smaller case series reported in the literature about the pregnancies complicated with AVB [3, 4, 7, 8]. Here, we present 74 cases of pregnant women with AVB. The cardiovascular and obstetric complications as well as the outcomes were analyzed and compared among patients with different grades of AVB.

## Methods

This was a retrospective study. Approval was obtained from the Institutional Review Board of West China Second University Hospital. We reviewed the medical records of 656 pregnant women with cardiac arrhythmias, who were admitted at West China Second University Hospital (the tertiary referral center) during the period from January 2010 to March 2020. Prevalence of atrioventricular block was 11.28% (74/656) in pregnancy with all cardiac arrhythmias.

All patients underwent clinical examination, ECG and transthoracic echocardiography, and fetal echocardiography. The following co-morbidities were taken into consideration in the clinical characterization of the patients: hypertension, diabetes mellitus, and other pregnancy associated diseases. Each pregnant woman in our center is individually analyzed to determine the delivery mode.

### Electrocardiogram

24-h ambulatory ECG was performed in patients who had abnormal electrocardiographic pattern (such as a slow heart rate) or normal ECG but had symptoms of dizziness, weakness, and syncope in early pregnancy.

### Echocardiography

All the patients were evaluated by echocardiography in order to screen other heart diseases. Fetal echocardiography also performed determine fetal heart disease.

### Other examinations/procedures

As necessary, vascular ultrasound was employed for diagnosing peripheral embolism. Cardiac ultrasound was also employed for excluded the presence of pulmonary hypertension. If a patient had signs of pulmonary hypertension such as tightness and cyanosis even if the patient was at rest, it needed to be jointly consulted by cardiologists and Intensive care physician, and then central venous catheterization (CVT) or right heart catheterization may be taken into account to monitor central venous pressure (CVP) and pulmonary artery pressure gradients according to the patient serious symptom.

### Statistical analysis

Descriptive statistical methods were used to represent variables, including frequency, percentage, mean, standard deviation (SD) and range. Chi-square test or Fisher’s exact probability test were used for comparing classification variables such as clinical features and complications. And the normal distribution variables were expressed by Means ± SD, the differences between groups were compared by Student t-test. The distribution of blood loss was presented as median ± IQR (interquartile range) and was compared using Mann–Whitney U test. SPSS (version 20.0) was used for all statistical analysis, and a *p*-Value of 0.05 was used as the cutoff for significance. This statistical analysis methods have also been described in our previous study for pregnancy complicated with tetralogy of Fallot [9].

## Results

According to the degree of atrioventricular block, the patients were divided into three groups: I° AVB (*n* = 16), II° AVB [*n* = 25; further divided into Mobitz type 1 (*n* = 18), type 2 (*n* = 7)], and III° AVB (*n* = 33).

### Patient characteristics

The sociodemographic information for these patients were summarized in the Table 1. Specifically, the overall age of patients ranged from 18 to 38 years old. The median of age was 30.6 years old in the group of first-degree AVB (I° AVB), 29.2 years old in the group of second-degree AVB (II° AVB), and 25 years old in the group of third-degree AVB (III° AVB). Interestingly, there had a different among the three groups. However, there was no significant difference in educational level (with vs without college degree) or living area (urban/suburban vs rural). The total number pregnancies (gravidity) and parity were greater in the groups of I° AVB and II° AVB than in the group of III° AVB.

**Table 1:** The sociodemographic information in patients with different degree of AVB

Patient characteristics	I° AVB (*n*=16)	II° AVB (*n*=25)	III° AVB (*n*=33)	*p*-Value
Age (years)				
Median age	30.62±4.67	29.17±4.46	25.45±3.92*	<0.001*
Living area				
Urban/Suburban	10	11	21	0.304
Rural	6	14	12	
College education				
Yes	11	16	15	0.199
No	5	9	18	
Gravidity				
First	4	13	31	<0.001^#^
Second	7	9	2	
Third or above	5	3	0	
Parity				
Primiparity	7	15	33	<0.001^▲^
Multiparity	9	10	0	

*Comparison of I ° AVB and III ° AVB, *p* = 0.000; Comparison of II °AVB and III °AVB, *p* = 0.001

^#^Comparison of I ° AVB and III ° AVB, *p* = 0.000; Comparison of II °AVB and III °AVB, *p* = 0.001

^▲^Comparison of I ° AVB and III ° AVB, *p* = 0.000; Comparison of II °AVB and III °AVB, *p* = 0.000

### Cardiac complications

Cardiac characteristics of the mothers are shown in Table 2. The cardiac function showed statistical significance among patient groups with different degree of AVB. Of note, it was found that cardiac function was significantly different between II° AVB type I and II° AVB type patients. Among the 33 patient in the III° AVB group, all patients except one were implanted with a pacemaker before delivery. Six of them (18.18%) were implanted with a permanent pacemaker before pregnancy. Twenty-six of them (78.79%) received temporary pacemaker before delivery. One patient with III° AVB did not receive pacemaker due to emergency cesarean section for premature delivery and fetal distress. During cesarean section, isoproterenol was pumped (1 mg/50 ml, 3 ml/h) to maintain the cardiac function. Because of abrupt drop of her heart rate she was transferred to cardiology department for pacemaker placement immediately after the cesarean section operation. In the group of II° AVB patients, two type I patients (11.11%) who were complicated with other bradyarrhythmias and NYHA class III-IV cardiac function received temporary pacemaker in the third trimester. The remaining II°AVB type I patients (88.89%) did not receive pacemakers. In contrast, most of patients with type II of II°AVB patients (85.7%) received pacemaker. Overall, pacemaker placement among the four groups of patients was statistically different.

**Table 2:** Clinical characteristics and cardiac complications in patients with different degree of AVB

Parameters	Total n (%)	I° AVB (*n*=16)	II° AVB (*n*=25)		III° AVB (*n*=33)	*p*-Value
			Type I (*n*=18)	Type II (*n*=7)		
NYHA-FC						
I - II	66 (89.2)	16 (100)	16 (88.89)	4 (57.2)	30 (90.91)	0.034**
III - IV	8 (10.8)	0	2 (11.11)	3 (42.8)	3 (9.09)	
Pacemaker implantation						
None	34 (45.95)	16 (100)	16 (88.89)	1 (14.3)	1 (3.03)^a^	<0.001^#^
Temporary pacemaker	34 (45.95)	0	2 (11.11)	6 (85.7)	26 (78.79)	<0.001
Permanent pacemaker	6 (8.1)	0	0	0	6 (18.18)	0.068
Left ventricular systolic function						
Normal	73 (98.65)	16 (100)	18 (100)	7 (100)	32 (96.97)	1.000
Dysfunction	1 (1.35)	0	0	0	1 (3.03)	
Valve condition						
Normal	66 (89.2)	14 (87.5)	16 (88.88)	5 (71.4)	31 (93.94)	0.304
AR	1 (1.35)	0	1 (5.56)	0	0	0.554
TR	3 (4.05)	0	0	1 (14.3)10	2 (6.06)	0.289
MR	2 (2.7)	1 (6.25)	0	1 (14.3)	0	0.049
TR+MR	2 (2.7)	1 (6.25)	1 (5.56)	0	0	0.389
With other arrhythmias						
None	35 (47.30)	11 (68.75)	1 (5.56)	0	23 (69.7)	<0.001^▲^
Sinus arrhythmia	13 (17.57)	1 (6.25)	7 (38.89)	3 (42.8)	2 (6.06)	0.003
Sinus tachycardia	5 (6.76)	1 (6.25)	2 (11.11)	1 (14.3)	1 (3.03)	0.388
Sinus bradycardia	3 (4.05)	0	1 (5.56)	0	2 (6.06)	1.000
APB	6 (8.11)	1 (6.25)	5 (27.77)	0	0	0.004
PVC	8 (10.81)	1 (6.25)	2 (11.11)	2 (28.6)	3 (9.09)	0.458
APB + PVC	4 (5.40)	1 (6.25)	0	1 (14.3)	2 (6.06)	0.334
With other heart abnormalities						
None	67 (90.54)	15 (93.75)	17 (94.44)	5 (71.4)	30 (90.91)	0.367
Left atrial enlargement	5 (6.76)	0	1 (5.56)	2 (28.6)	2 (6.06)	0.135
Pericardial effusion	2 (2.7)	1 (6.25)	0	0	1 (3.03)	0.780

*N/n* number, *AVB* Atrioventricular block, *NYHA-FC* Cardiac function grading (New York Heart Association), *AR* Aortic regurgitation, *MR* mitral regurgitation, *TR* tricuspid regurgitation, *APB* atrial premature beat, *PVC* premature ventricular contraction

^a^One patient with III° AVB did not install pacemaker before emergency cesarean section due to premature delivery and fetal distress. During the operation, isoproterenol was pumped (1 mg/50ml, 3 ml/h), and then the patient was transferred to cardiology department for pacemaker placement after the operation immediately

** Comparison of I° AVB and Type II, *p*=0.020;

# Temporary pacemaker: Comparison of (I° AVB+ Type I) and (Type II +III° AVB), *p*<0.001; Comparison of I° AVB and III° AVB, *p*<0.001

▲With other arrhythmias and none, comparison of III° AVB and II° AVB, *p*<0.001

The Left ventricular systolic function was in normal range in the majority of studied patients, except one patient with III° AVB. There was no significant difference in the condition of heart valve among the four groups of patients, though MR condition showed a *p*-value < 0.05, which may be due to small number of case (only two patients). While 35 patient (47.3%) had AVB alone, more than half of patients (52.7%) were also complicated with other types of arrhythmias. Of note, the other types of arrhythmia were mainly observed among II° AVB patients. More or less other arrhythmias were also found in other groups. However, only sinus arrhythmia and APB showed statistical difference among the four groups of patients, the other arrhythmias had no significant differences. Regarding other heart abnormalities, left atrial enlargement and pericardial effusion were observed in all groups of patients with no significant difference.

### Obstetric complications

The clinical data of obstetric complications including fetal complication and maternal complications are summarized in the upper part of Table 3. Obstetric complications were found in 21 women (28.4%). Because of two cases of monochorionic diamniotic twin (MCDA), the total of 76 fetuses were observed in 74 mothers.

**Table 3:** Obstetric complications and outcome of pregnancies in patients with different degree of AVB

Complications and Outcome	No(%) (M=74/F=76)^a^	I° AVB (M=16/F=17)	II° AVB(*n*=25)		III° AVB (*n*=33)	*p*-value
			Type I (M=18/F19)	Type II (*n*=7)		
Fetal complications						
Prematurity	11 (14.47)	1 (5.88)	6 (31.58)	1 (14.29)	3 (9.09)	0.116
SGA	2 (2.63)	0	1 (5.26)	1 (14.29)	0	0.099
Fetal distress	1 (1.32)	0	0	0	1 (3.03)	1.000
Oligohydramnios	2 (2.63)	1 (5.88)	1 (5.26)	0	0	0.398
Maternal complications						
PROM	3 (4.05)	1 (6.25)	0	0	2 (6.06)	0.707
GDM	3 (4.05)	0	1 (5.56)	0	2 (6.06)	1.000
MCDA	2 (2.70)	1 (6.25)	1 (5.56)	0	0	0.389
Preeclampsia	1 (1.35)	0	0	0	1 (3.03)	1.000
Placental adhesion	6 (8.11)	0	3 (16.67)	1 (14.29)	2 (6.06)	0.220
Velamentous placenta	1 (1.35)	0	1 (5.56)	0	0	0.554
Thrombocytopenia in pregnancy	3 (4.05)	1 (6.25)	1 (5.56)	0	1 (3.03)	1.000
proteinuria + pedal edema	1 (1.35)	0	0	1 (14.29)	0	0.095
Mode of delivery						
Caesarean section	60 (81.08)	10 (62.5)	12 (66.67)	7 (100)	31 (93.94)	0.009^#^
Vaginal delivery	14 (18.92)	6 (37.5)	6 (33.33)	0	2 (6.06)	
Fetal cardiac abnormalities						
Fetal TR	1 (1.32)	1 (5.89)	0	0	0	0.316 1.000
Fetal VSD	2 (2.63)	0	1 (5.26)	0	1 (3.03)	
Transfer to ICU	4 (5.45)	1 (6.25)	0	2 (28.57)	1 (3.03)	0.053
Transfer to cardiology	2 (2.70)	0	0	0	2 (6.06)	0.780
Neonatal death	0	0	0	0	0	-
Maternal death	0	0	0	0	0	-
Postpartum hemorrhage	3 (4.05)	0	0	1 (14.29)	2 (6.06)	0.289
Mean neonatal weight (kg)	76	3148.23±435.93(*n*=17)	3101.05±531.61(*n*=19)	3278.57±545.81	3026.21±320.23	0.479
HOD (day)	74	4.37±1.14	4.44±1.14	5.28±1.60	4.87±0.89	0.160
Blood loss during delivery (ml)	74	353.12±88.44	511.11±234.86	507.14±73.19	575.75±167.29	0.001^▲^

*M* Maternal, *F* Fetal, *SGA* Small for gestational age, *PROM* Premature rupture of membranes, *GDM* Gestational diabetes mellitus, *MCDA* Monochorionic diamnionic twin, *TR* Tricuspid Regurgitation, *VSD* Ventricular septal defect, *ICU* Intensive care unit, *HOD* Hospital day (total days of hospital stay)

^a^Total number of fetuses was 76 because there are two patients with twin pregnancies (maternal number = 74); *Two case with fetal heart hyperechoic both have been checked by high-throughput genome sequencing, and showed no abnormality in prenatal screening;

^#^Multiple test results: comparison of I ° AVB and III ° AVB, *p* = 0.017; Type I AVB and III ° AVB, *p*= 0.031

^▲^Multiple test results: comparison of I ° AVB and type I AVB, *p* = 0.008; I ° AVB and type II AVB *p* = 0.047; I ° AVB and III ° AVB, *p*= 0.000

The most common fetal complications related to the maternal AVB were prematurity, which was observed in 11 fetuses (14.47%). In addition, we observed two cases (2.63%) of oligohydramnios, two cases of small for gestational age (SGA, in which the estimated infant weight after delivery below the 10th percentile for reference gestational age), and one case (1.32%) of fetal distress. The maternal complications, including premature rupture of membranes (PROM), gestational diabetes mellitus (GDM), preeclampsia, placental adhesion, velamentous placenta, thrombocytopenia in pregnancy, proteinuria + pedal edema, were reviewed and analyzed. None of these maternal complications showed significant difference among the four groups of patients. Other obstetric conditions frequently encountered in normal pregnancy, such as nuchal cord (9/76, 11.84%) and breech presentation (2/76, 2.63%), thromboembolism (0, 0%) were also reviewed, none of them appeared to be more concerning in AVB patients than in general population.

### Mode of delivery

Among the studied cases, 60 (81.08%) women underwent caesarean section and 14 (18.92%) women had vaginal delivery. As shown in the middle section of Table 3, cesarean section was performed in 100% (7/7) II° AVB type II and 93.94% (31/33) III° AVB patients, the vaginal delivery mainly occurred in I° AVB and II° AVB type I patients.

### Maternal and perinatal outcomes

There was no maternal or neonatal death in our patients. And no difference for postpartum hemorrhage, mean neonatal weight, hospital day among the four groups of patients. But the blood loss during delivery was significantly differents (*P* < 0.05). Among the 76 fetuses, fetal cardiac abnormalities were observed in 3 cases (3.95%). Prenatal screening with genome sequencing was performed for two cases of hyperechoic fetal heart, and no abnormality was found.

Most of patients with AVB recovered uneventfully and no complication in the postpartum period in our study. Just one patient with I° AVB and two patient with II° AVB type II transferred to ICU, and two patients with III° AVB transfer to cardiology department. The patient with I° AVB suffered repeated heart rate drop (the lowest heart rate of 35 beats/min) on the second day after operation transfer to ICU and was given medical treatment such as atropine and isoprenaline. One patient with II° AVB type II was not implanted with a temporary pacemaker before deliver, postpartum ECG showed frequent ventricular premature beats and induced supraventricular tachycardia. Another one patient with II° AVB type II was placed the temporary pacemaker before the birth, but the patient had edema of lower limbs and urine protein (with normal blood pressure) during her 3rd trimester, there was no relief after the birth and progress to acute left heart failure with symptoms of rapid breathing difficulties and orthopnea, and the distribution of scattered wheeze of lungs could be heard. Two patients transfer to cardiology department for permanent pacemaker placement after the operation immediately all were the patients with III° AVB (one patient did not install pacemaker before emergency cesarean section due to premature delivery and fetal distress, and another one was implanted with temporary pacemaker before operation).

## Discussion

Atrioventricular block (AVB) is caused by the dysfunction of the cardiac electroconductive pathways and characterized by a prolongation of the PR interval on the electrocardiogram (ECG) [10]. According to the pattern and feature of the disruption of electrical activity, the blocks are classified in three categories: first degree, second degree (Mobitz type 1 or 2), and third-degree [10, 11]. The pathogenesis underlying the block could be an anatomical defect or functional impairment in the heart’s conduction system [11].

The high-grade heart block can cause palpitations, fatigue, dyspnea, and/or syncope, and associated with significant mortality [4]. Importantly, during the laboring process, bradycardia may get worse when uterine contractions displace blood into the central circulation. The serious cardiac and obstetric complications may occur and impact both mother and fetus. In the literature, only some case reports for pregnancy with AVB have been found, and there is no an established consensus guiding peripartum management for pregnant women with AVB [3–8]. It will be beneficial to study and share our experiences with these pregnant women.

### Maternal cardiac complications

In general, with the increase in the degree of AVB, the complications and mortality of patients increased. Most pregnant patients with I° AVB were asymptomatic and did not have significant complications. While the treatment was not necessary for these patients, regular evaluation is essential because the risk of atrial fibrillation and development of higher degree AVB increases with the growth of gestational age [1]. In our study, no patient in this group needs the placement of a pacemaker.

Regarding II° AVB, the block may be temporary or permanent depending on the impairment of the conduction system and divided into two types (Mobitz type I and Mobitz type II). Patients with II° AVB may be asymptomatic or have symptoms like syncope and lightheadedness [1]. Of note, Mobitz type II block has the potential for progressing to a complete heart block, which may result in death if unrecognized. Previous study reported that a patient with type II of II° AVB identified at 24 gestational weeks. She had symptoms of palpitations and fatigue and experienced episodes of asystole, and the longest episode lasted for 15.8 s [12]. In our study, other mild arrhythmias were also frequently present in the patient with II° AVB type I AV block. But the severe arrhythmias including PVC and PVC + APB were observed in second-degree type II AV block. Therefore, patients with II° AVB type II have high risk for more unexpected complications, which may change and progress rapidly during pregnancy and lead to adverse maternal and neonatal outcomes.

The III° AVB indicates a complete loss of communication between the atria and the ventricles, so it’s also called complete heart block. Without appropriate conduction through the AV node, the SA node cannot control the heart rate, and cardiac output can diminish secondary to the loss of coordination between the atria and the ventricles. The condition can be fatal if not promptly treated. Most patients usually receive a temporary pacemaker and later replaced by a permanent one. Pacemaker can improve the survival rate of patients with III° AVB during pregnancy [1, 8]. In this study, except one patient who did not have a pacemaker because of emergency surgery, the remaining 32 patients with III° AVB received pacemaker implantation before delivery and did not have cardiac/obstetric complications or maternal death.

In the literature, several studies demonstrated that pregnancy in women with high-grade AVB was associated with high maternal and fetal mortalities, if pacemaker placement could not be achieved before delivery [8, 13, 14]. However, it has also been proposed that temporary or permanent pacemakers in asymptomatic women should be assessed on a case-by-case basis [6, 9]. One recent study found that stress tests to assess the change between the PR interval and heart rate could help to identify those higher-risk individuals and could be used as possible indication for the placement of a permanent pacemaker [15]. In our center, the patients complicated with heart disease have been managed conservatively by a multidisciplinary team. In the current study, permanent pacemakers have been placed in 6 patients with severe symptoms before pregnancy, and all of them maintained good cardiac function during pregnancy. The remaining patients have been closely monitored during pregnancy and received temporary pacemakers (except the above mentioned one) before delivery and removed after delivery. The good NYHA classification and the pacemaker placement may be relevant factors for maternal and neonatal outcomes. In addition, these extensive care and intervention might help the patients with high-grade AVB to achieve the similar overall outcome as in the patients with I° AVB.

### Congenital atrioventricular block and fetal cardiac abnormalities

Atrioventricular block could be congenital or acquired post birth. Congenital AVB may be caused by cardiac malformations or damaged by maternal antibodies (autoimmune AVB). Family history of AVB is one of the risk factor for congenital AVB [16, 17]. During pregnancy of patients with autoimmune conditions, maternal autoantibodies can across the placenta and attack the atrioventricular (AV) node in susceptible fetuses. For example, anti-Ro/SS-A is one specific type of antinuclear auto antibodies, which have been found to be associated with fetal congenital heart disease (CHB) [16–18]. It has been reported that one woman with congenital heart disease and III° AVB gave birth to one child with CHB [19]. In our study, cardiac abnormalities were identified in 3 out of 76 fetuses (3.95%), which was slightly higher than among fetuses (3.23%) born by pregnant women with tetralogy of Fallot who our research group have studied previously [19]. Based on the close relationship between anti-Ro/SS-A antibodies and AVB and the family history, we were interested in studying the autoimmune antibodies in pregnant women with AVB. Unfortunately, auto antibodies are not routinely tested for pregnant women in our hospital, we could not retrieve the data to study anti-Ro/SS-A antibodies in pregnant women with AVB in the current study. It will be studied in our future research to test this assumption.

### Obstetric complication

In our study, 28.4% patients showed obstetric complications, including premature labor, premature rupture of membranes (PROM), gestational diabetes mellitus (GDM), preeclampsia, placental adhesion, velamentous placenta, thrombocytopenia in pregnancy, proteinuria. The most common fetal complications were prematurity, oligohydramnios, SGA and fetal distress. No neonatal and maternal death was observed in the current study.

For pregnant women with AVB, in addition to obstetric conditions, the baseline cardiac function and the risk for exacerbation of cardiac function during delivery were also taken into the consideration. In the current study, while most patients (81.08%) with high NYHA-FC and/or high-grade AVB received caesarean section, 14 women (18.92%, including 2 patients with III° AVB) had successful vaginal delivery. Of note, all patients with II° AVB type II received cesarean section. Two of our patients with III° AVB who have received pacemaker showed good outcome for the vaginal delivery. Accordingly, the presence of a pacemaker in a patient may not necessarily be an indication for cesarean section [3]. The determination of delivery mode is not straightforward for pregnant women with AVB, evaluation from multidisciplinary team may be necessary to achieve the best benefit for both mother and neonate.

In summary, most women with AVB could achieve successful pregnancy and delivery. Patients with II° AVB type II and III° AVB should be monitored vigilantly during pregnancy and post-partum. Temporary pacing before delivery appeared to be beneficial for women with III° AVB. In the patients without pacing, especially those with II° AVB type II should be closely monitored with precautious plan for unexpected complications.

## Data Availability

The dataset supporting the conclusions of this article is included within the article.

## Abbreviations

AVB: Atrioventricular block
I°AVB: First-degree AVB
II°AVB: Second-degree AVB
III°AVB: Third-degree AVB
SA node: Sinoatrial node
AV node: Atrioventricular node
ECG: Electrocardiography
CVT: Central venous catheterization
CVP: Central venous pressure
SD: Standard deviation
N/n: Number
NYHA-FC: Cardiac function grading (New York Heart Association)
AR: Aortic regurgitation
MR: Mitral regurgitation
TR: Tricuspid regurgitation
APB: Atrial premature beat
PVC: Premature ventricular contraction
M: Maternal
F: Fetal
SGA: Small for gestational age
PROM: Premature rupture of membranes
GDM: Gestational diabetes mellitus
MCDA: Monochorionic diamniotic twin
VSD: Ventricular septal defect
ICU: Intensive care unit
HOD: Hospital day (total days in hospital)
